# Muehrcke's lines in a psoriatic patient: A possible association with acitretin therapy

**DOI:** 10.1002/ccr3.2434

**Published:** 2019-10-07

**Authors:** Fatemeh Mohaghegh, Farzaneh Danesh

**Affiliations:** ^1^ Department of Dermatology School of Medicine Isfahan University of Medical Sciences Isfahan Iran

**Keywords:** acitretin, leukonychia, Muehrcke's line, nail

## Abstract

Muehrcke's lines are a type of apparent leukonychia that are common in patients receiving chemotherapeutic agents. They are self‐limited, and there is no need to more workup.

## Graphical abstract

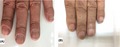

## 1 BACKGROUND

Two transverse white lines parallel to lunula in nail bed, which is associated with chronic hypoalbuminemia states such as nephrotic syndrome, are called Muehrcke’s lines.^1^ They were first reported in 1956 by Muehrcke.^2^ These lines may transiently vanish following the administration of pressure to the distal digits. Here in this paper, we report a case of unusual Muehrcke's lines following acitretin therapies for psoriasis. Although the exact mechanism of Muehrcke's lines is still unknown, these lines are considered as a valuable sign of hypoalbuminemia and are mostly associated with metabolic stress and sometimes with chemotherapy drugs.^3^ Muehrcke's lines are highly associated with administration of chemotherapeutic agents and mostly resolve after drug discontinuation. To the best of our knowledge, only one case of Muehrcke's lines following acitretin therapies has been reported.

## 2 CASE PRESENTATION

### 2.1 Case 1

**Figure 1:**
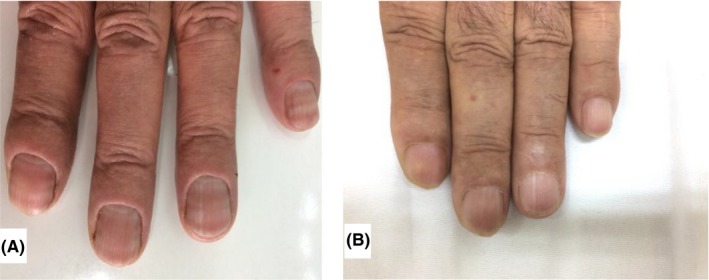
Muehrcke's line in psoriasis patient. A, During treatment with acitretin. B, 2 months after discontinuous of drug

A 54‐year‐old man who was a previously known case of psoriasis treated with topical corticosteroid and emollients was presented with fingernail involvement as nail dystrophy and erythema, scaling, and pustules around the fifth fingernail of right hand that did not respond to topical therapies. There was no significant point in the past medical history. The laboratory data including renal function test, liver function test, and serum albumin levels were normal. Treatments with acitretin 25 mg daily were initiated. After 5 months of treatments, paired transverse white lines were noted involving all the fingernails and made patient anxious. On each finger, there were two pale lines 1 mm in width, parallel to the lunula with regular border. The lines were disappeared with pressing on the nail plate, and they did not move distally during nail growth (Figure 1A).

After 2 months of discontinuation of acitretin, patient represented with white lines faded (Figure 1B).

## 3 DISCUSSION AND CONCLUSIONS

Firstly, Muehrcke described a nail condition as two parallel lines of pallor in the nail bed in the patients with low serum albumin,^4^ now known as Muehrcke’s lines. Muehrcke attributed this physical finding to “chronic nutritional deficiency of albumin”.^5^

Muehrcke’s lines are double white band parallel to the lunula^6^ that spans the entire nail bed. Applying the pressure on distal digits results in fading of these lines.^6^ During the nail growth, they remain fixed and do not migrate distally.^7^ The pathogenesis is not completely known but may be caused by compression on the vessels of nail bed by local edema.^5^

The most important differential diagnosis is Mee’s line in which there is disturbance in nail plate but not in nail bed.^8^ Also, we can easily distinguish it from true leukonychia because it fades with compression.^9^

Muehrcke's lines could be present in the absence of hypoalbuminemia.^5^ They are very common in patients receiving chemotherapeutic agents,^10^ most commonly after treatment with anthracycline, cyclophosphamide, and vincristine and usually occur 3‐4 weeks of the use of agent.^11^ There is a case report on Muehrcke's lines induced by transretinoic acid therapy, an analogue of vitamin A, in patients with acute promyelocytic leukemia.^8^ But, here we presented a case of Muehrcke's lines following acitretin therapies which is vitamin A analogues. There has also been a case report of a 58‐year‐old woman with transverse leukonychia due to acitretin treatments for palmoplantar pustular psoriasis^12^ published in 2013. This case is also similar to our case. The probable pathogenesis in the setting of chemotherapy is alteration of nail plate attachment to the nail bed due to vascular abnormalities due to chemotherapy.^1^ Taken together, Muehrcke's lines can be associated with different clinical and pathological conditions and mostly will resolve after drug discontinuation or dietary support. In this paper, we published for the first time, a case of Muehrcke's lines associated with acitretin treatments which faded after drug discontinuation.

This condition is asymptomatic and resolves after drug withdrawal.^9^ So, we should reassure the patient about the benign nature of it and there is no need for more workups or referral to other specialists.

## CONFLICT OF INTEREST

The authors declare that they have no competing interests.

## AUTHORS' CONTRIBUTIONS

FM: designed and collected the data and wrote, read, and approved the final manuscript. FD: collected the data and wrote, read, and approved the final manuscript.

## ETHICS APPROVAL AND CONSENT TO PARTICIPATE

This study does not require any approval from the ethics committee.

## CONSENT FOR PUBLICATION

The authors have consent for publication.
